# Two COX-2 inhibitors induce apoptosis in human erythroleukemia K562cells by modulating NF-κB and FHC pathways

**DOI:** 10.1186/s40199-015-0139-0

**Published:** 2016-01-07

**Authors:** Shaghayegh Norouzi, Mahnaz Norouzi, Mohsen Amini, Amir Amanzadeh, Mohamad Nabiuni, Saeed Irian, Mona Salimi

**Affiliations:** Department of Cell and Molecular Biology, Faculty of Biological Sciences, Kharazmi University, P.O. Box 1481765544 Tehran, Iran; Department of Medicinal Chemistry, School of Pharmacy, Tehran University of Medical Sciences, Tehran, Iran; National Cell Bank of Iran, Pasteur Institute of Iran, Tehran, Iran; Department of Physiology and Pharmacology, Pasteur Institute of Iran, P.O. Box 13164 Tehran, Iran

**Keywords:** Leukemia, Apoptosis, COX-2, FHC, NF-κB

## Abstract

**Background:**

Leukemia is distinguished by abnormal proliferation of leukocytes. Although there has been some progress in developing novel cancer therapies, no significant improvement was observed in the overall survival rate over the last decade. Selective cyclooxygenase-2 (COX-2) inhibitors are known to inhibit tumor growth by exerting antimetastatic and antiangiogenic effects through inhibition of COX –dependent and independent pathways. The ability of two new triaryl-oxadiazole derivatives, compounds **A** (3-(4-chlorophenyl) -5-(4-flurophenyl)-4-Phenyl-4,5-dihydro-1,2,4-oxadiazole) and **B** (3,5-bis(4-chlorophenyl)-4-Phenyl-4,5-dihydro-1,2,4-oxadiazole), to induce apoptosis in human erythroleukemia K562 cells was evaluated and the upstream mechanism was investigated.

**Methods:**

K562 cells were treated with compounds **A** and **B** at their IC_50_ concentrations and analyzed by DAPI staining and Annexin-V-FLUOS labelling solution. Nuclear factor kappa-B (NF-κB) activation was evaluated by TransAM kit. Cyclooxygenase-2 (COX-2), Caspase-3, Bax, Bcl-2, ferritin heavy chain (FHC), extra cellular signal-regulated kinase (ERK), p-ERK and early growth response protein-1 (Egr1) levels were determined using Western blotting, while c-Myc mRNA level was investigated by RT-PCR.

**Results:**

Changes in nuclear morphology and the increased annexin-V/PI staining revealed the apoptotic cell death in compounds **A-** and **B**-treated K562 cells. A significant reduction in NF-κB activity as well as FHC and p-ERK levels were detected in these cells. No change was observed in the levels of Bax, Bcl-2, Caspase-3, COX-2, c-Myc and Egr1, following treatment with the two compounds. Collectively, compounds **A** and **B** potentiate apoptosis as shown by DAPI staining, flowcytometry, FHC and p-ERK downregulation and NF-κB inactivation.

**Conclusion:**

Two compounds induce apoptosis in a COX-2-independent manner which also appears to be independent from mitochondria, caspase and c-Myc/Egr1 pathways.

## Background

Leukemia, a cancer of the body’s blood-forming tissues, including the bone marrow and the lymphatic system, is distinguished by abnormal proliferation of leukocytes. Based on the International Classification of Childhood Cancer, leukemia represents one of the largest diagnostic groups among individuals under 15 years of age with incidence of 34 % [1]. Although there has been some progress in developing novel cancer therapies, no significant improvement was observed in the overall survival rate over the last decade [2]. Nonsteroidal anti-inflammatory drugs (NSAIDs) with their pain relief and anti-inflammation properties have also been the focus of attention as anti-cancer agents [3]. The targets of traditional NSAIDs are cyclooxygenases 1 and 2 (COX-1 and COX-2), enzymes involved in the production of prostaglandins from arachidonic acid [4]. In this regard, NSAIDs are known to inhibit tumor growth by exerting antimetastatic and antiangiogenic effects through inhibition of COX activity, however, a COX-independent pathway has also been suggested [3, 5].

In addition to common NSAIDs, the newly developed selective COX-2 inhibitor, celecoxib, with a better gastrointestinal risk profile, has been considered as a cost-effective alternative [6]. Celecoxib has been proven as a potent candidate for treating cancer, with several ongoing clinical trials as well as in various animal tumor models [5, 7]. Celecoxib has also been demonstrated to have inhibitory effect on the growth of K562 cells, and induce apoptosis [5, 8].

**Fig. 1:**
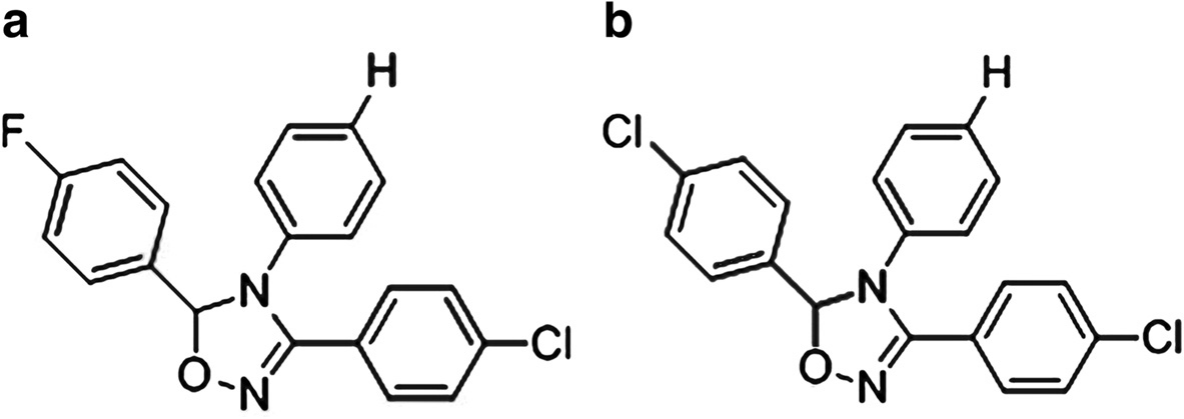
Structure of the two new celecoxib derivatives

Celecoxib represents a 1, 2-di-aryl heterocyclic structure and used as an ideal lead compound for developing novel derivatives with potent apoptosis-inducing activity [9, 10]. We have recently reported that two compounds with triaryl-oxadiazole structures known as compounds **A** (3- (4-chlorophenyl) -5-(4-flurophenyl)-4-Phenyl-4,5-dihydro-1,2,4-oxadiazole) and **B** (3,5-bis(4- chlorophenyl)-4-Phenyl-4,5-dihydro-1,2,4-oxadiazole) (Fig. 1) show significant biological features such as antiproliferative activity with considerable IC_50_ values (21.66 and 22.23 μM) in human erythroleukemia (K562) cell line after a 24 h treatment [11]. In the present investigation, we examined the mechanism leading to apoptosis during treatment of K562 cell line with the two new celecoxib derivatives, compounds **A** and **B**.

## Methods

### Drugs and reagents

Compounds **A** and **B** were synthesized by the Department of Medicinal Chemistry, Tehran University of Medical Science (Tehran, Iran). Dulbecco’s Modified Eagle’s Medium (DMEM) and fetal bovine serum (FBS) were purchased from Gibco-BRL (Rockville, IN, USA). Annexin-V-FLUOS kit was prepared from Roche Applied Science (Indianapolis, USA). Polyclonal anti–caspase-3 (1:500), anti-Bcl-2 (1:500), anti-Bax (1:500), anti-COX-2 (1:1000), anti-GAPDH (1:1000) antibodies and monoclonal anti-ERK (1:1000), anti-Phospho-ERK (1:1000), anti-FHC (1:100) and anti-Egr-1 (1:200) antibodies were purchased from Abcam (Cambridge MA, USA). Anti-rabbit IgG horseradish peroxidase (HRP) antibody (1:5000) was obtained from Cell Signaling Technology (Beverly, MA, USA). All other chemicals were in high purity and prepared from Merck (Darmstadt, Germany).

### Cell culture

K562 cells were obtained from the cell bank of Pasture Institute of Iran (NCBI). Cells were cultured in DMEM medium containing 10 % FBS, 100 U/mL penicillin and 100 μg/mL streptomycin. These cells were incubated at 37 °C and 5 % CO2 in a humidified atmosphere and then were treated with compounds **A** and **B** at the IC_50_ concentrations (21.66 and 22.23 μM) for 8 and 16 h.

### Analysis of cell morphology by DAPI staining

The treated and untreated cells were stained by DAPI 4’,6-diamido-2-phenylindole hydro chloride) (Roche Applied Science, Indianapolis, USA), and their morphology was observed under a Zeiss fluorescence microscope (Zeiss, Germany). Photomicrographs were taken with an Olympus digital camera (Tokyo, Japan).

### Identification of apoptosis by Annexin-V/PI staining

Following treatment, 10^6^ cells were washed in PBS and resuspended in 100 μL of annexin-V-FLUOS labeling solution containing 2 μL annexin-V-FLUOS labeling agent, 2 μL Propidium Iodide (PI) solution and 1 mL incubation buffer to achieve a concentration of 10^6^ cells/mL. Following incubation at 37 °C for 15 minutes, cells were analyzed by flowcytometry. Annexin-V binds to cells expressing phosphatidyl serine on the outer layer of the cell membrane, and PI stains the cellular DNA of those with a compromised cell membrane [12]. This allows for the discrimination of live cells (unstained with either fluorochrome or PI) from apoptotic (stained with annexin-V) and necrotic cells (stained with PI).

### Western blotting

K562 cells were treated with compounds **A** and **B** at their IC_50_ concentrations for 16 h. Proteins were extracted from distinctively treated cells, collected and lysed in lysis buffer (Tris 62.5 mM (pH 6.8), DTT 50 mM, SDS 10 %, glycerol) in the presence of protease inhibitors. Then, equal amounts of protein were heated to 95 °C, separated in 12 % SDS-polyacrylamide gels and transferred to PVDF membranes. The membrane was then blocked for 2 h in TBST (50 mmol/L Tris-Cl, pH 7.6, 150 mmol/L NaCl and 0.1 % Tween 20) containing 1 % (w/v) casein, and then incubated with primary antibodies overnight, followed by incubation with HRP conjugated goat anti-rabbit IgG for 2 h. Blots were then developed using ECL advance western blotting detection kit. The signals from each protein band were normalized against the GAPDH (Glyceraldehyde Phosphate Dehydrogenase) content using the polyclonal anti-GAPDH antibody. The expression level of control was designated value “1”, and thereby the expression ratios of the treatments were expressed in relation to the control.

### ELISA-based TransAM assay

K562 cells were treated with compounds **A** and **B** at their IC_50_ concentrations for 16 h. Proteins of treated cells were extracted. The experiments were performed according to manufacturer’s instructions for the ELISA-based TransAM p65 kit (TransAM NF-κB p65 TranscriptionFactor Assay kit). Briefly, 20 μg of protein extract per well was added to a 96-well plate coated with the immobilized oligonucleotide containing the NF-κB consensus sequence (5’-GGGACTTTCC-3’) and incubated for 1 h at room temperature with mild agitation. p65 protein present in the extract will specifically bind to this sequence. Next, anti-p65 antibody (100 μL, at a 1:1000 dilution) was added to each well and incubated for 1 h, followed by the addition of 100 μL of horseradish peroxidase (HRP)-conjugated antibody (1:1000 dilution) for yet another 1 h. After adding 100 μL of the developing solution for up to 5 min, colorimetric reaction was stopped and ELISA reader was used to quantify the difference between the intensity of NF-κB p65 by reading absorbance at 450 nm with a correction wavelength of 630 nm.

### Semiquantitative RT-PCR

For analysis of c-Myc mRNA expression levels, cells were treated or not treated with compounds **A** and **B** for 16 hours, and total RNA was extracted with RNX-Plus (CinnaGen, Iran) following the manufacturer’s protocol. 1 μg of total RNA was used to perform reverse transcription with the RevertAid first strand cDNA synthesis Kit (Thermo, Lithuania) following the manufacturer’s protocol. The resulting reverse transcription products were stored at −70 °C until use.

Reaction products were quantified by RT-PCR using a 2X TaqPreMix (Master Mix) PCR Kit (CinnaGen, Iran) according to the manufacturer’s instructions. PCR of GAPDH that was chosen as an internal control was carried out in the same tubes as for the genes. The PCR primers of GAPDH and c-myc were synthesized according to the references [13, 14]. Sequences of the primers were as follows: c-myc (Forward: 5’- TGGTGCTCCATGAGGAGACA-3’; Reverse: 5’-GTGTTTCAACTGTTCTCGTC-3’), GAPDH (Forward: 5’-GAGCCCGCAGCCTCCCGCTT-3’; Reverse: 5’- CCCGCGGCCATCACGCCACAG-3’).

### Statistical analysis

Each experiment was performed at least three times and representative data were shown. Data in the graph are given as mean values ± standard error (SE) of the mean. Significant differences among groups were determined using a one-way ANOVA followed by the posttest tukey.

## Results

### Apoptosis induction in K562 cells following compounds A and B treatments

**Fig. 2:**
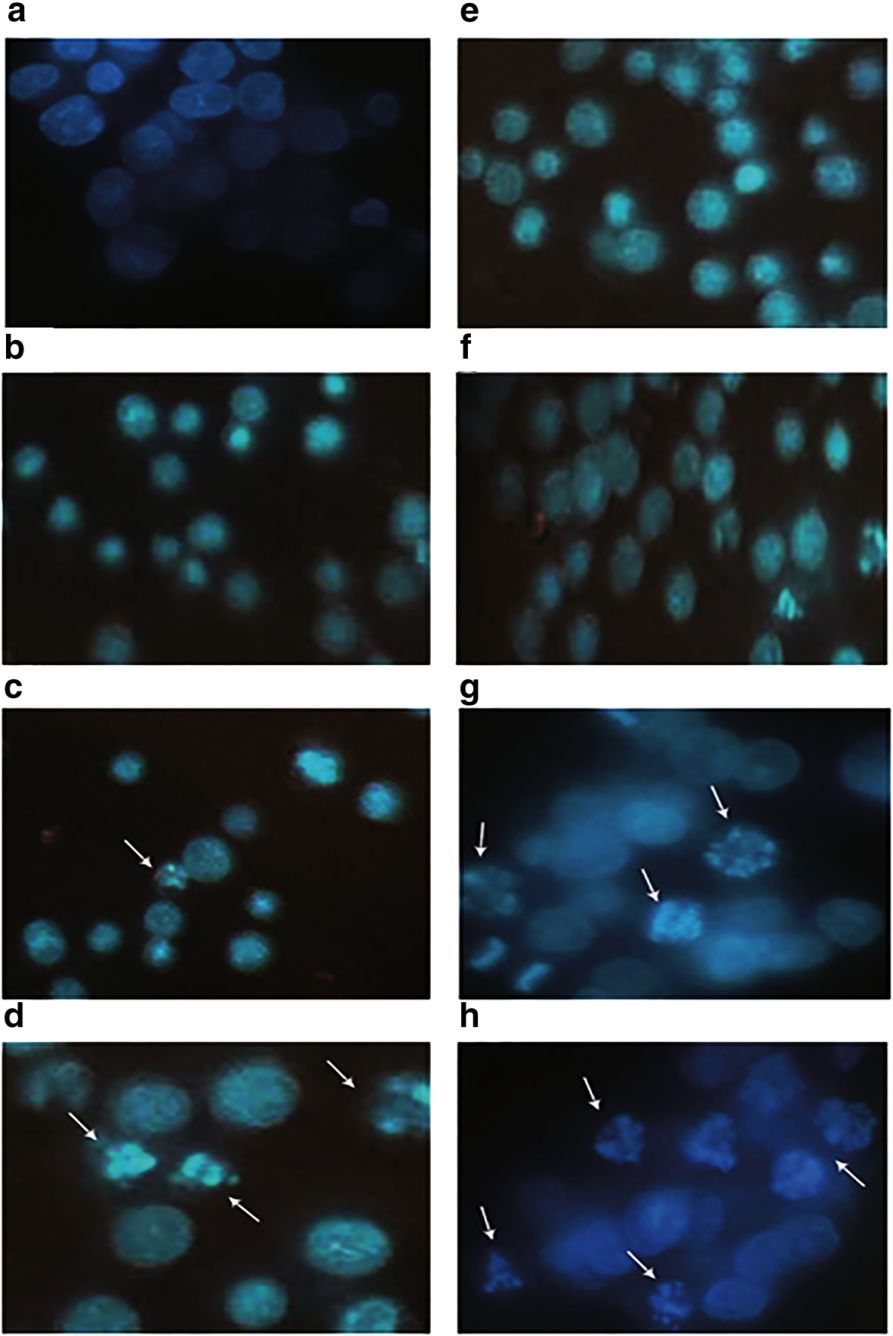
Apoptotic morphological changes in K562 cells. **a** K562 control cells after 8 h. **b** K562 cells treated with DMSO for 8 h. **c** K562 cells treated with compound A at 21.66 μM for 8 h. **d** K562 cells treated with compound B at 22.23 μM for 8 h. **e** K562 control cells after 16 h. **f** K562 cells treated with DMSO for 16 h. **g** K562 cells treated with compound A at 21.66 μM for 16 h. **h** K562 cells treated with compound B at 22.23 μM for 16 h. Cells were stained with DAPI and observed by fluorescent microscopy

In order to examine whether compounds **A** and **B** exhibit cytotoxicity in K562 cells through apoptosis, DAPI staining analysis was undertaken in order to observe morphological changes in K562 cells following treatment with compounds **A**(21.66 μM) and **B**(22.23 μM) for 8 and 16 h (Fig. 2). Morphological evidence of apoptosis was detected as chromatin condensation and nuclear fragmentation following 8 h (Fig. 2d) and to a greater extent at 16 h (Fig. 2h) treatment with compound **B**, whereas compound **A**-treated K562 cells showed slight morphological changes at 8 h (Fig. 2c) and to a greater extent at 16 h (Fig. 2g).

**Fig. 3:**
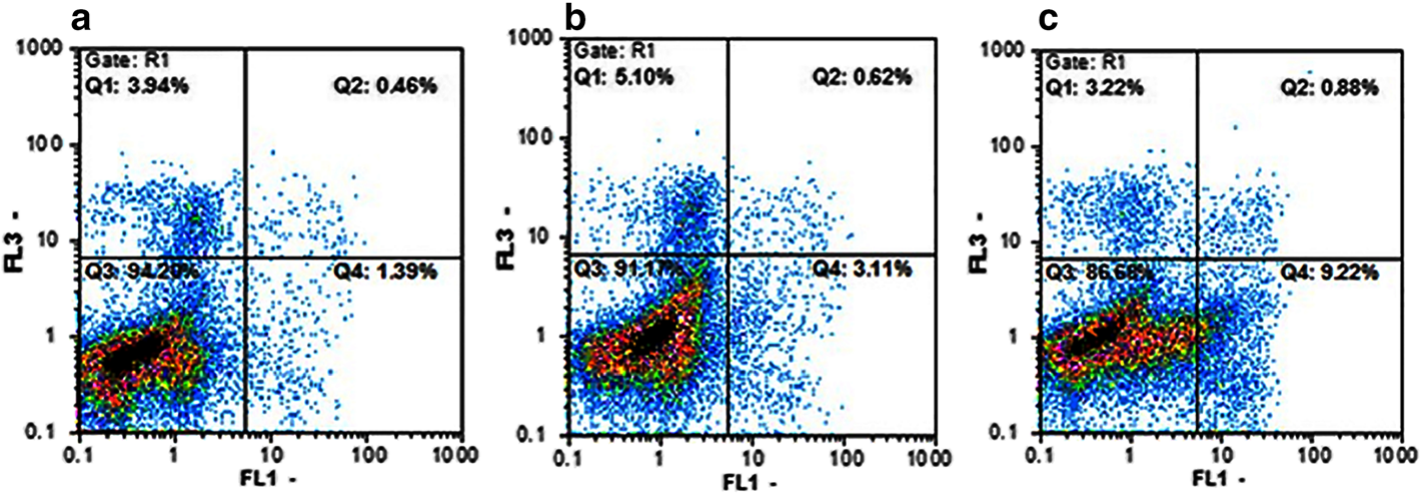
Flowcytometry analysis to quantify apoptosis in K562 cells. **a** Untreated control K562 cells. **b** Cells treated with compound A (21.66 μM). **c** Cells treated with compound B (22.23 μM). Cells were stained with annexin V and propidiumiodide. The results shown are representative of three independent experiments. Quadrant 3, living cells An−/PI−; Quadrant 4, early apoptotic cells An+/PI−; Quadrant 2, late apoptotic cells An+/PI+; Quadrant 1, necrotic cells An−/PI+

Apoptosis induced by compounds **A** and **B** in K562 cells was confirmed by staining cells with Annexin-V/PI and subjecting them to flowcytometry. In the dual parameter fluorescent dot plots, the number of cells in early (annexin V+/PI−, the lower right quadrant) and late (annexin V+/PI+, the upper right quadrant) apoptosis were counted (Fig. 3). As shown in Table 1, 95.02 % of untreated cells were viable. However, treatment with compounds **A** (IC_50_) and **B** (IC_50_) for 16 h resulted in a reduction in the number of viable cells to 90.48 % and 85.95 %, respectively, whereas, the percentage of annexin V+/PI− cells (early apoptosis) increased significantly. These results demonstrate that compounds **A** and **B** show antitumor activities in K562 cells, mainly through the induction of apoptotic cell death.

**Table 1:** Percentage of K562 cells in each state after treatment with compounds A (21.6 μM) and B (22.23 μM) for 16 h

Compound	Vital cells (%) An–/PI–	Early apoptosis (%) An+/PI–	Late apoptosis (%) An+/PI+	Necrosis (%) An–/PI+
A	90.48±0.38	0.427497	0.61±0.09	4.9±0.23
B	85.91±0.62	9.82±0.58	1.05±0.02	3.22±0.08
Control	95.02±1.1	1.28±0.06	0.36±0.05	3.34±0.4

### Effect of compounds A and B on Bax, Bcl-2 and caspase-3 levels

**Fig. 4:**
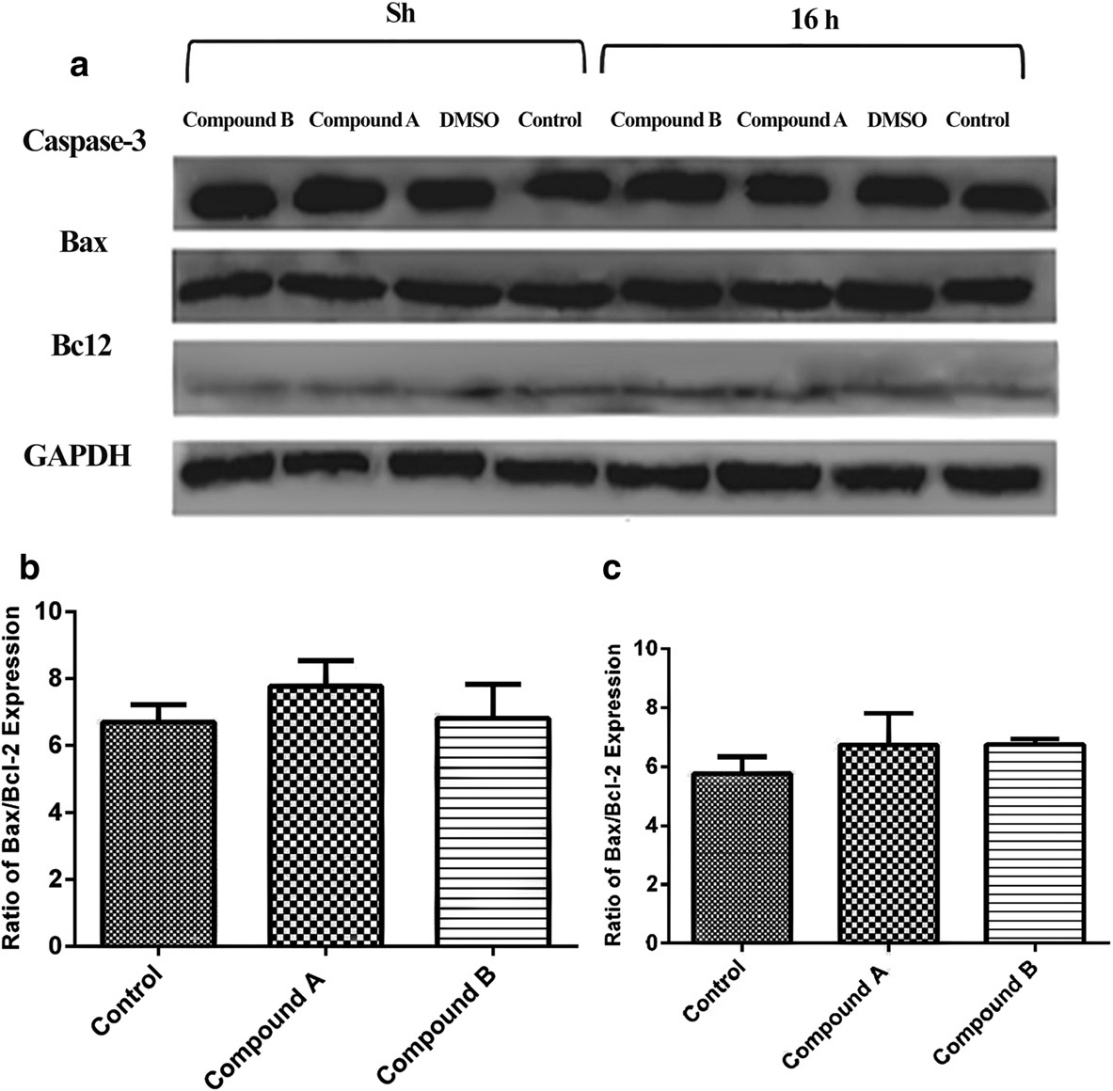
Expression analysis of apoptosis associated proteins by Western blot analysis. K562 cells were treated with compounds A (21.66 μM) and B (22.23 μM). **a** Immunoblot showing the expression levels of caspase-3, Bcl-2, Bax and GADPH. **b** Ratios of Bax/Bcl-2 after 8 h. **c** Ratios of Bax/Bcl-2 after 16 h. The results of 3 independent experiments are presented as mean ± standard error

In order to determine the possible involvement of the caspase cascade in the execution phase of apoptosis, caspase-3 level was investigated following treatment of cells with the IC_50_ concentration of compounds **A** and **B** by Western blot analysis. As shown in Fig. 4a, treatment with either compound had no effect on the cleavage of caspase-3 in K562 cell line after 8 and 16 h. In addition, the expression of Bcl-2 and Bax, measured by Western blot analysis, revealed no apparent change in Bax/Bcl-2 ratio at 8 and 16 h (Fig. 4b and c).

### Effect of compounds A and B on COX-2 levels

**Fig. 5:**
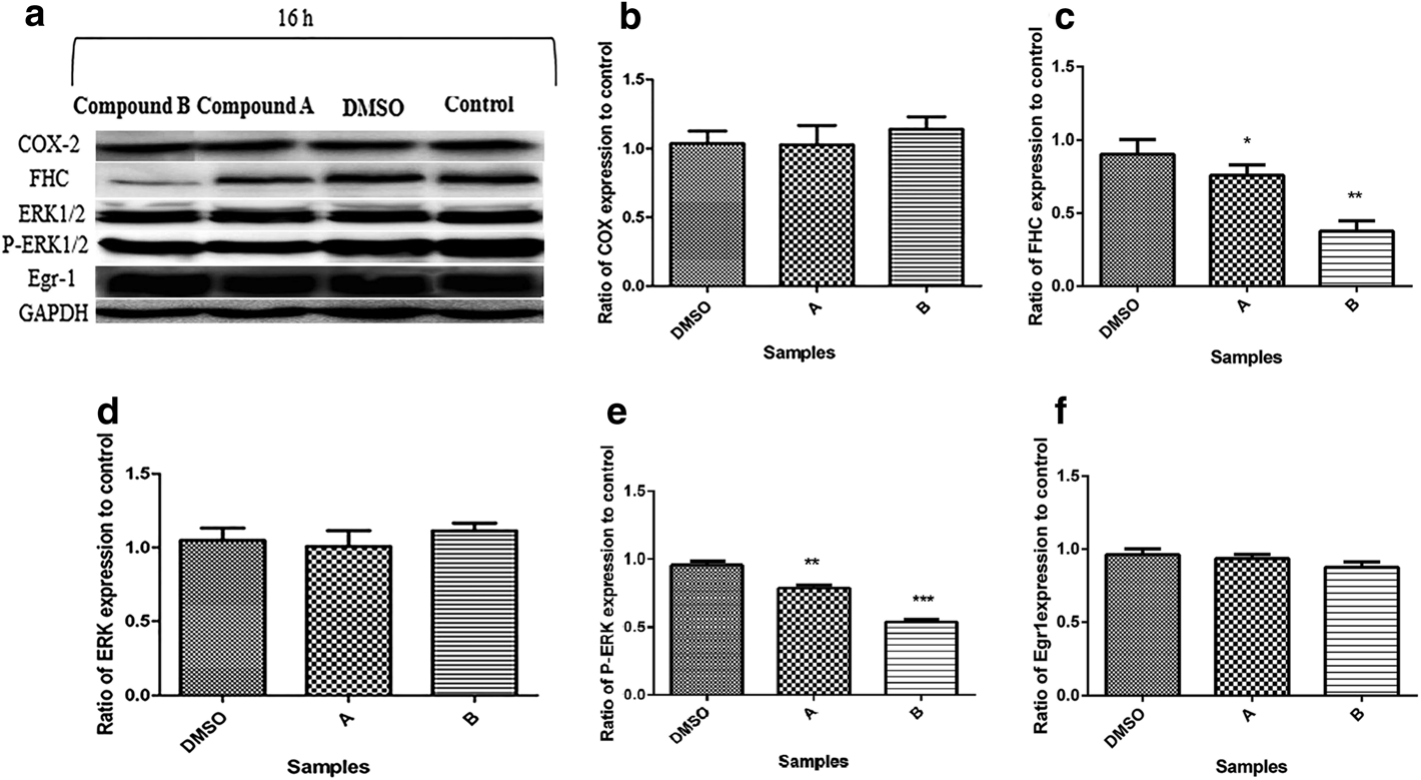
Effect of compounds **A** (21.6 μM) and **B** (22.23 μM) on COX-2, FHC, ERK1/2, p-ERK1/2 and Egr1 expression in K562cells after incubation for 16 hours. **a** Immunoblot showing the expression levels of COX-2, FHC, ERK1/2, p-ERK1/2, Egr1 and GAPDH. **b** Ratios of COX-2/control. **c** Ratios of FHC/control. **d** Ratios of ERK/control. **e** Ratios of p-ERK/control. **f** Ratios of Egr1/control. The results of 3 independent experiments are presented as mean ± standard error (*P < 0.05, **P < 0.01 vs. the solvent group)

In order to determine the possible involvement of the COX-2 pathway in apoptosis induced by compounds **A** and **B**, COX-2 level was investigated following treatment of cells with the IC_50_ concentrations of the two compounds. As shown in Fig. 5a and b, neither of the compounds had any significant effect on the expression of COX-2 following a 16 h treatment of K562 cells.

### Effect of compounds A and B on NF-ĸB activity

**Fig. 6:**
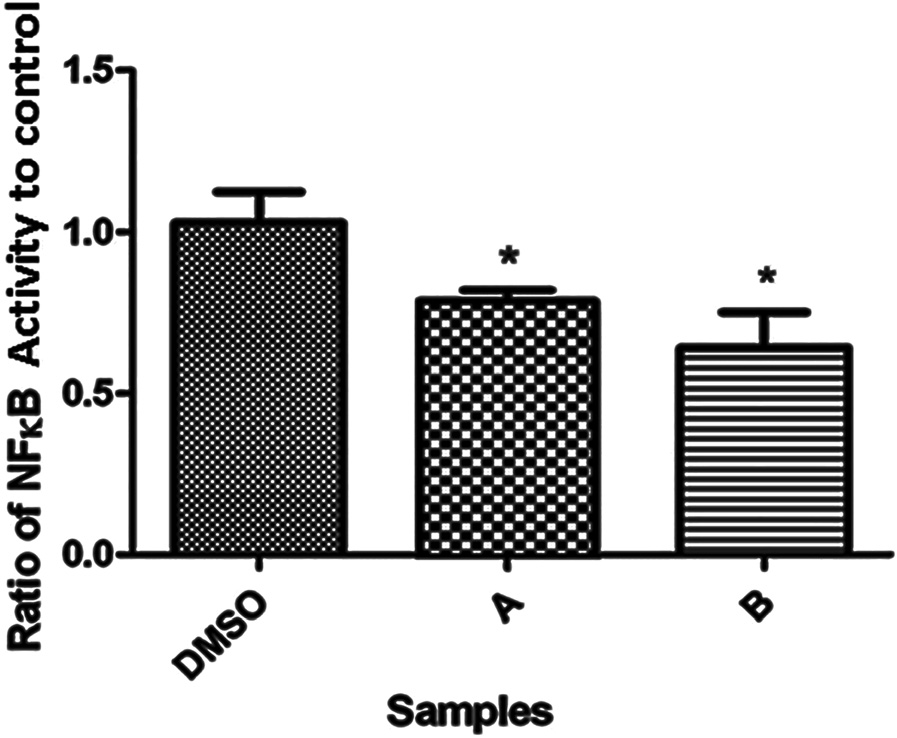
Effect of compounds A (21.6 μM; IC_50_) and B (22.23 μM; IC_50_) on NF-κB activation in K562 cells after incubation for 16 hours. The results of 3 independent experiments are presented as mean ± standard error (*P < 0.05, vs. the solvent group)

As NF-ĸB plays a critical role in the apoptosis process [15], the effects of compounds **A** and **B** on NF-ĸB p65 activity was assessed. As shown in Fig. 6, following a 16 h treatment of cells with compounds **A** and **B**(IC_50_), a significant (*p* < 0.05) reduction in the concentration of nuclear p65 subunit was observed.

### Effect of compounds A and B on FHC, p-ERK, c-Myc and Egr1 levels

**Fig. 7:**
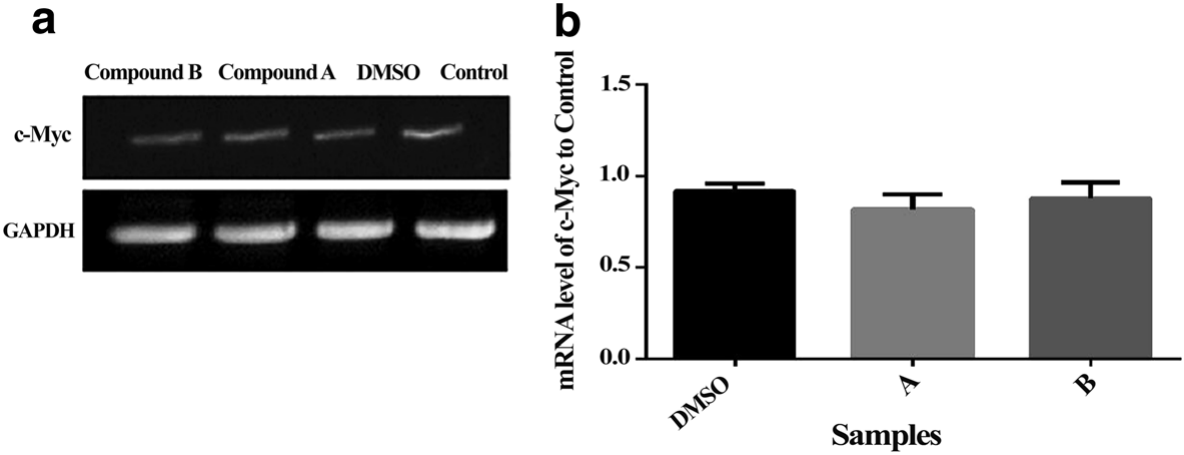
Effect of compounds A (21.6 μM) and B (22.23 μM) on c-Myc mRNA levels in K562 cells after incubation for 16 hours. **a** Immunoblat showing the expression level of c-Myc. **b** Ratios of c-Myc/control. The results of 3 independent experiments are presented as mean ± standard error

To reveal upstream mechanism of apoptosis induction following treatment with celecoxib derivatives, levels of certain factors, located downstream of NF-ĸB in the apoptosis signaling pathway, including FHC, p-ERK, c-Myc and Egr1 were determined through Western and RT- PCR analysis (Fig. 5 and 7).

Administration of compounds **A** and **B** to K562 cells resulted in a reduction in FHC protein level, which was significant (*p* < 0.01) following 16 h of treatment (Fig. 5a and c). Treatment of cells with the two compounds **A** and **B** also revealed a significant (*p* < 0.01 and *p* < 0.001, respectively) reduction in the levels of p-ERK which coincided with a lack of change in those of ERK after 16 h of treatment (Fig. 5a, d and e). Collectively, these results demonstrate that treatment with compounds **A** and **B** lead to a reduction in both ERK phosphorylation and FHC levels. Finally, Semi-quantitative RT-PCR analysis revealed no change in c-Myc mRNA levels in cells treated with compounds **A** or **B** (IC_50_) after 16 h (Fig. 7). Furthermore, consistent with the transcript levels of c-Myc, protein levels of Egr1 were not altered by compounds **A** and **B** after 16 h (Fig. 5a and f).

## Discussion

To study the cytotoxicity of compounds **A** and **B**, as two new triaryl-oxadiazole derivatives which well fitted to COX-2 active site [11], their effects on the induction of “programmed cell death” in K562 cells, and the mechanisms by which these compounds induce apoptosis were investigated. K562 cells are a human cancer blood cell line derived from the chronic myeloid leukemia (CML) tumor lineage, which has been considered as a good model to study the cancer inhibitory effects of celecoxib derivatives [5, 8]. Celecoxib, as a first generation of COX-2 inhibitors, has gained a great deal of attention in cancer studies. Although, the cardiotoxic effect has been established in long period treatment of patients with celecoxib, and for this reason, its application in chemoprevention has been restrained [16]. In this regard, the two new compounds **A** and **B** show significant antiproliferative activity with considerable IC_50_ values (21.66 and 22.23 μM) in K562 cell line after a 24 h treatment [11].

The main mechanism that attributes to the observed effects of COX-2 inhibitors against different types of cancer is apoptosis [17]. Apoptosis is a kind of cell death known as “programmed cell death” that occurs in different physiological and pathological situations and marked by typical morphological and biochemical features, including cell shrinkage, nuclear DNA fragmentation and membrane blebbing [18]. Following treatment of K562 cells with compounds **A** and **B** for 16 h, the fluorescence microscopic observations demonstrated typical morphological changes indicating cell damage; however, these changes were not as much following 8 h of incubation (Fig. 2). The data obtained from annexin-V/PI analysis were in agreement with the morphological changes showing that compounds **A** and **B** induce apoptosis after 16 h (Table 1).

The two major apoptotic pathways known to operate in mammalian cells include the receptor (extrinsic) and the mitochondrial (intrinsic) pathways. Through the intrinsic pathway, members of the Bcl-2 protein family operate as regulators of survival and death during apoptosis induction [19, 20]. Although the role of mitochondria in compounds **A**- and **B**-induced apoptosis is not clear in K562 cells, several cytotoxic drugs as well as COX-2 inhibitors are known to initiate apoptosis through the mitochondrial pathway [21]. Our data clearly demonstrate that Bax and Bcl-2 do not have a critical role in the induction of apoptosis in K562 cells (Fig. 4), suggesting a mitochondria-independent apoptotic pathway induced by compounds **A** and **B**. The role of caspase-3 in the mitochondria-independent apoptotic pathway has also been well understood [22]. However, apparently compounds **A** and **B** induce apoptosis in a caspase- independent mechanism as caspase-3 levels were not altered by compounds **A** and **B** in K562 cells (Fig. 4a). Collectively these data demonstrate that compounds **A** and **B** induce apoptosis through both a mitochondria- and caspase- independent mechanism in K562 human leukemia cells. Our obtained data are in agreement with the recent report indicating the execution of apoptosis without the involvement of caspases. Indeed, apoptosis induced in these cells by compounds **A** and **B** has been shown to involve the activation of proteases other than caspases [23].

The antiproliferative and apoptotic effects of celecoxib in K562 cells are associated with COX-2 inhibition [5]. However, it is well established that tumor growth inhibition by selective COX-2 inhibitors might as well be mediated through COX-2-independent mechanisms [24]. In this study, treatment with compounds **A** and **B** were found to cause no change in COX-2 levels (Fig. 5). These findings demonstrate that the apoptotic effects of these compounds in K562 cells are mediated by a COX-2 independent pathway. Interestingly, it was recently reported that celecoxib derivatives with no COX-2 inhibitory action can be considered as anti-cancer agents without increasing the cardiovascular risk [25]. Therefore, the two compounds may have a potential in being used as chemotherapeutic agents with little or no cardiovascular side effects.

Induction of apoptosis can be triggered by a number of stimuli, activating a cell suicide program that is constitutively present in most vertebrate cells. In diverse cell types, Rel/NF-kappaB transcription factors are involved in regulation of apoptosis by either inducing or perhaps more commonly blocking it [26]. NF-κB regulates the expression of more than 400 genes involved in inflammation, cell survival, proliferation, invasion and angiogenesis. NF-κB also affects apoptosis during tumor development and progression [27], often leading to apoptosis resistance [28]. On the other hand, a reduction in NF-κB mitogenic signaling has been associated with the anticancer effects of celecoxib treatment on mouse mammary epithelial tumor cells mediated through a COX-2 independent pathway [24]. Our data also demonstrate that treatment of K562 cells with compounds **A** and **B** results in a reduction of NF-κB expression (Fig. 6) in a COX-2-independent manner.

The primary iron storage factor, FHC (Ferritin heavy chain), which mediates the antioxidant and protective activities of NF-κB, is induced downstream of NF-κB and required for TNFα-induced apoptosis [29]. Rezaie et al. (2011) reported FHC down-regulation in J774.A1 macrophage-like cell lines, following celecoxib treatment [30]. Our study confirms down-regulation of FHC expression induced by compounds **A** and **B** in K562 cell line (Fig. 5). Interestingly, our data exhibit the down regulation of both NF-κB and FHC which may not be surprising as NF-κB, as a transcription factor, has been involved in the regulation of FHC expression.

Moreover, mitogen-activated protein kinase (MAPK)/extracellular signal-regulated protein kinase (ERK) have been implied in transmitting the apoptotic signal [14]. MAPK family members are mediators of signal transduction pathways [22]. Importantly, NF-ĸB acts the upstream of MAPKs in the signaling of certain anticancer drugs [31]. The ERK pathway, a major player in the regulation of cell growth, survival, and differentiation, is induced in response to mitogens and growth factors [22], however, evidence for ERK pathway mediating apoptosis has also been reported [32]. According to previous studies, celecoxib has different roles in ERK1/2 activation in different cells. The present study revealed growth inhibition of K562 cells following compounds **A**- and **B**- treatment via down regulation of p-ERK1/2 (Fig. 5). These data indicate that the mechanism of anti-proliferative activities of the designed compounds may be mediated through NF-ĸB and ERK inhibition.

Anticancer drugs induce apoptosis through different intracellular targets and cause DNA damage [21]. Another factor contributing to the regulation of apoptosis includes the early gene, c-Myc [33]. There has been conflicting reports on the regulation of c-Myc expression during NSAID-induced apoptosis. NSAID-induced apoptosis of gastric cancer cells may be mediated through the up-regulation of c-Myc proto-oncogene [34], while NS-398, a COX-2 inhibitor, reduces c-Myc expression in rat colon cancer cells [35]. In the present study, RT-PCR analysis revealed no alteration in the levels of c-Myc transcript in K562 cells treated with compounds **A** or **B** compared to the control (Fig. 7b), however, compounds **A** and **B** induced apoptosis in K562 cells, suggesting the operation of a c-Myc independent apoptotic pathway. Our results are consistent with those of Ostrowski et al. (2003) who demonstrated a lack of alteration in basal and serum-stimulated c-Myc expression following treatment of rat hepatoma HTC-IR cells with aspirin or celecoxib. In addition, when treated with the selective COX-2 inhibitor, NS-398, no change was observed in c-Myc mRNA levels in rat colon mucosa as compared to untreated animals [36].

Previous studies also showed that c-Myc directly induces transcription of a noncanonical target gene, Egr1 (Early growth response-1) [37]. Egr1 can suppress the growth of a number of tumor cells, including leukemia. A diverse of anticancer agents, such as COX inhibitors, act through the modulation of Egr1 [38]. In a pancreatic cancer model, tolfenamic acid-induced Egr1 expression appears to have an essential role in the activation of apoptosis [39]. Our data revealed no change in the levels of Egr1 in K562 cell line treated with compounds **A** and **B** (Fig. 5). Therefore, the apoptosis in K562 cell line induced by compounds **A** and **B** appears to be independent from the c-myc/Egr1 pathway. These results are in line with our previous report showing an apoptosis induced by compounds **A** and **B** via a c-myc/Egr1 independent pathways in breast cancer cell line with a different mechanism [40].

## Conclusion

In summary, the present study reveals that compounds **A** and **B**, as two new triaryl-oxadiazole derivatives with COX-2 inhibitory structures, induce apoptosis in K562 cells through a mitochondrial-, caspase- and COX-2-independent pathways. This COX-2-independent mechanism involves a reduction in NF-κB levels and a subsequent reduction in the downstream FHC and P-ERK1/2 signaling. Our findings also demonstrate a more pronounced apoptotic effect in response to compound **B** than compound **A** in K562 cells. Future studies need to be directed towards identifying the downstream mediators in order to reveal a more clear and detailed molecular pathway that would help in the development of compounds **A** and **B** as suitable chemotherapeutic agents against leukemia.
